# Analysis of *MET* mRNA Expression in Gastric Cancers Using RNA In Situ Hybridization Assay: Its Clinical Implication and Comparison with Immunohistochemistry and Silver In Situ Hybridization

**DOI:** 10.1371/journal.pone.0111658

**Published:** 2014-11-03

**Authors:** Jiwoon Choi, Hee Eun Lee, Min A. Kim, Bo Gun Jang, Hye Seung Lee, Woo Ho Kim

**Affiliations:** 1 Department of Pathology, Seoul National University College of Medicine, Jongno-gu, Seoul, Korea; 2 Department of Pathology, Seoul National University Bundang Hospital, Gyeonggi, Korea; CCR, National Cancer Institute, NIH, United States of America

## Abstract

We investigated *MET* mRNA expression status using RNA in situ hybridization (ISH) technique in primary and metastatic lesions of 535 surgically resected gastric carcinoma (GC) cases. We compared the results with those of immunohistochemistry and silver in situ hybridization, and examined the association with clinicopathologic characteristics and prognosis. Among 535 primary GCs, 391 (73.1%) were scored 0, 87 (16.3%) were scored 1, 38 (7.1%) were scored 2, 12 (2.2%) were scored 3 and 7 (1.3%) were scored 4 by RNA ISH. High *MET* mRNA expression (score ≥3) was associated with lymph node metastasis (*P* = .014), distant metastasis (*P* = .001), and higher TNM stage (*P*<.001). *MET* mRNA expression was correlated with protein expression (r = 0.398; *P*<.001) and gene copy number (r = 0.345; *P*<.001). The patients showing high-*MET* mRNA in primary or metastatic lesions had shorter overall survival than those showing low-*MET* mRNA (primary tumors, *P* = .002; metastatic lymph nodes, *P*<.001). The patients showing positive conversion of *MET* mRNA status in metastatic lymph node had shorter overall survival than those with no conversion (*P* = .011). Multivariate analysis demonstrated that high *MET* mRNA expression in metastatic lymph node was an independent prognostic factor for overall survival (*P* = .007). Therefore, this study suggests that *MET* mRNA expression assessed by RNA ISH could be useful as a potential marker to identify *MET* oncogene-addicted GC.

## Introduction

During the past decade, receptor tyrosine kinase (RTK) pathways have proven to be attractive drug targets for anticancer therapy [1], and the MET pathway is one of these promising targets. *MET* is a proto-oncogene located on the 7q31 locus and encodes an RTK for hepatocyte growth factor (HGF) [2], [3]. The tight regulation of the HGF/MET pathway that is observed in development and regeneration is lost in cancer, and such deregulation occurs through multiple mechanisms [1]. Aberrant MET activation plays important roles in cancer cell survival, growth, angiogenesis, and metastasis in various cancers including lung, breast, kidney, and gastrointestinal tract malignancies [4]. However, although patient stratification according to MET expression or activity is important for therapeutic success, the methods for assessing the level of MET expression or activity have not been established [4].

For gastric carcinoma (GC), aberrant MET activation has been thought to be related to a gene dosage effect [5], and *MET* gene amplification (GA) or protein overexpression has been associated with aggressive tumor characteristics and/or worse clinical outcome [6]–[14]. Furthermore, *MET*-amplified or -overexpressed GC showed response to treatment with several inhibitors of the HGF/MET signaling pathway in preclinical studies [15] and phase I clinical trials [12], [16]. Hence, MET inhibition has the potential as another successful therapeutic strategy following human epidermal growth factor receptor 2 (HER2)-targeted therapy in advanced GC. However, previous studies have used various methods to identify MET-positive GC and have shown discrepancy in the prevalence of MET overexpression or amplification: MET overexpression ranged 18% to 73.7% in studies using immunohistochemistry (IHC) [7]–[9], [13], [14], [17], *MET* gene copy number (GCN) gain ranged 10% to 21.2% in studies using quantitative real-time polymerase chain reaction (qPCR) [10], [11], and *MET* GA ranged 2% to 3.9% in studies using fluorescence in situ hybridization (FISH) [12], [18] or silver in situ hybridization (SISH) [13]. Of these methods, IHC is widely used in clinical practice and the most likely screening method for detection of MET-positive GC. However, further exploration is still needed to find a predictive biomarker or assay methodology for MET inhibition therapy.

In this study, we performed an RNA in situ hybridization (ISH) assay using paired DNA oligonucleotide probes and preamplifier-amplifier-label probes for visualization [19]. This method uses formalin-fixed, paraffin-embedded (FFPE) tissues and allows single-molecule visualization under a bright-field microscope. In our previous study, we proved that *HER2* mRNA expression evaluated by RNA ISH was well correlated with protein overexpression and GA evaluated by IHC and FISH in 211 GC cases [20]. Also, we showed the correlation between *MET* GCN and protein expression in a previous study [13]. Here, we evaluated *MET* mRNA expression using RNA ISH method, and compared the results with those of IHC and SISH in a large series of GC. In addition, clinicopathologic parameters and clinical outcomes of GC patients according to *MET* mRNA expression status were evaluated.

## Materials and Methods

### Patients and tissue specimens

We collected archival tissue samples of GC patients who consecutively underwent gastrectomy at Seoul National University Hospital, Seoul, Korea, from January 2004 through December 2005. Finally, 535 samples of primary GC and 199 samples of synchronous regional metastatic lymph node (LN) from 535 patients were available for this study. The clinicopathologic characteristics of the patients were examined by reviewing medical charts and pathologic records (Table 1). TNM stage was classified according to the system of the American Joint Committee on Cancer Staging Manual, 7^th^ edition. Clinical outcomes were followed up from the date of surgery until death or 60 months.

**Table 1:** Demographic and clinical characteristics of 535 gastric carcinoma patients.

Characteristics	
Median age (range), y	60 (24–87)
Gender, n (%)	
Male	368 (68.8)
Female	167 (31.2)
Tumor location, n (%)	
Upper third	53 (16.3)
Middle third	90 (27.6)
Lower third	170 (52.1)
Tumor histology and differentiation, n (%)	
Tubular/Papillary ADC, WD	37 (6.9)
Tubular/Papillary ADC, MD	179 (33.5)
Tubular/Papillary ADC, PD	206 (38.5)
Signet ring cell carcinoma	83 (15.5)
Others	30 (5.6)
Lauren classification, n (%)	
Intestinal	238 (44.5)
Diffuse	209 (39.1)
Mixed/indeterminate	88 (16.4)
Radicality. n (%)	
R0	499 (93.3)
R1/R2	36 (6.7)
Adjuvant chemotherapy, n (%)	
No	234 (43.7)
Yes	301 (56.3)
TNM stage, n (%)	
I	170 (31.8)
II	142 (26.5)
III	175 (32.7)
IV	48 (9.0)

Abbreviations: ADC, adenocarcinoma; MD, moderately differentiated; PD, poorly differentiated; TNM, Tumor-Node-Metastasis; WD, well differentiated.

All tissue samples were fixed in 10% buffered formalin for 24–48 hours and then embedded in paraffin. Core tissues (2 mm in diameter) were taken using a trephine apparatus (Superbiochips Laboratories, Seoul, Korea). For the primary GCs, the invasion front of each primary tumor was selected. Metastatic LNs were subjected to the tissue array but the cases with micrometastasis were excluded. Total 22 tissue microarray blocks which contained up to 60 cores were constructed.

### Ethical statement

All human specimens were obtained during therapeutic surgery. The participants did not provide the written consent to participate in this study. The retrospective study was performed using the samples over the shelves after the pathologic diagnosis, and all of the samples were anonymized before the study. Our IRB (Seoul National University Hospital) approved this retrospective study under the condition of the anonymization (Reference: H-1006-035-320).

### RNA ISH

**Figure 1:**
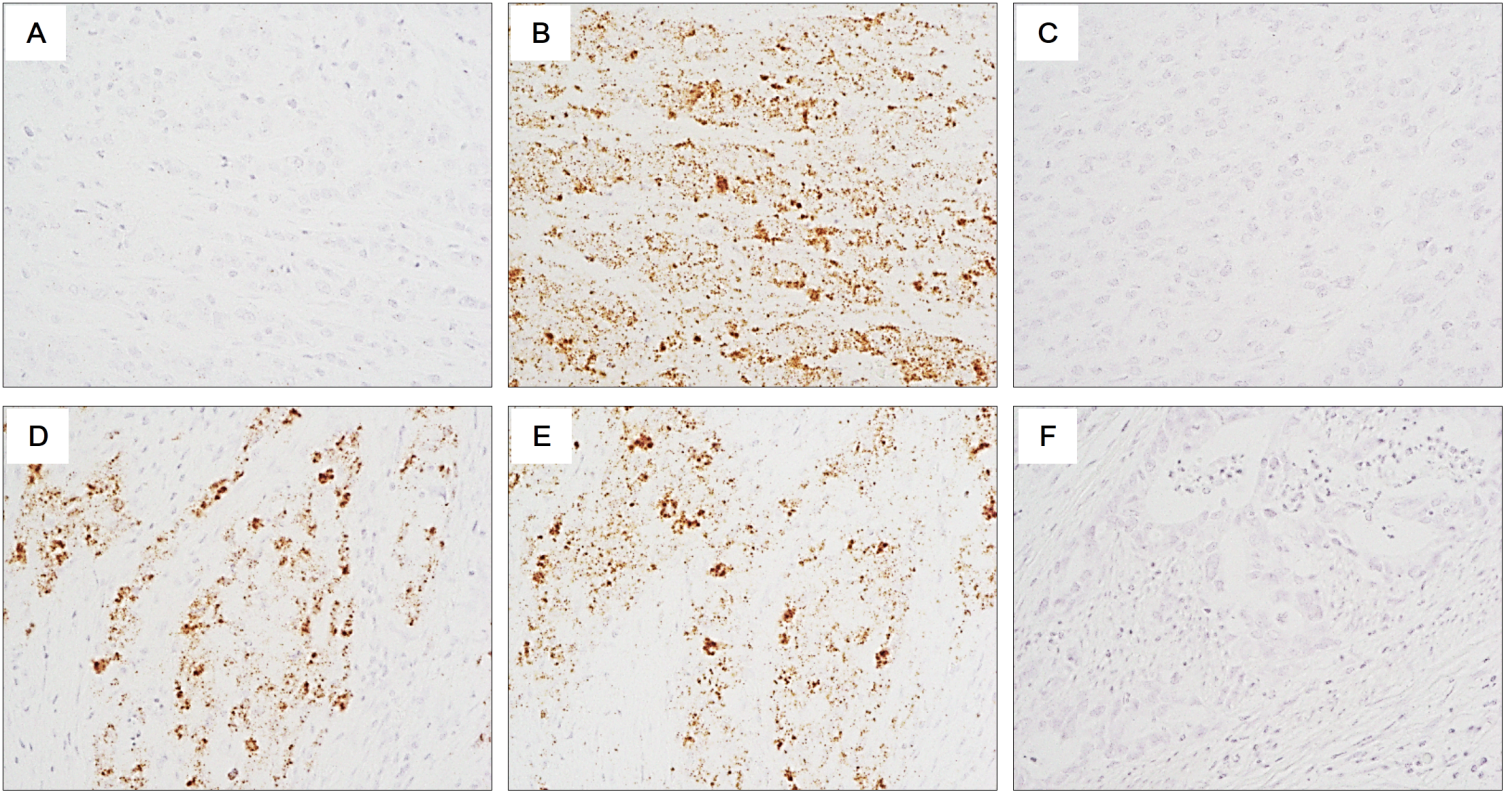
Representative figures of RNA in situ hybridization (ISH). (A–C) a negative case showing *MET* RNA ISH score 0: (A) *MET* mRNA, (B) *UBC* mRNA, and (C) *dapB* mRNA. (D–F) a positive case showing MET RNA ISH score 4: (D) *MET* mRNA, (E) *UBC* mRNA, and (F) *dapB* mRNA (original magnification: ×400).

For in situ detection of *MET* mRNA, the RNAscope FFPE 2.0 assay kit (Advanced Cell Diagnostics, Hayward, CA, USA) was used according to the manufacturer’s instructions. Briefly, 2- to 3-µm thick FFPE tissue sections were deparaffinized, heated, treated by protease, and hybridized with probe at 40°C for 2 hours (the reference sequence, NM_001127500; probe region, 1236–2257). After washing and amplification, 3, 3′-diaminobenzidine was added for detection of target RNA. Nuclei were counterstained with hematoxylin. Positive staining was indicated by brown punctate dots in the nucleus and/or cytoplasm. *MET* mRNA expression levels were categorized into 5 grades according to the manufacturer’s scoring guideline: score 0, no staining or <1 dot per cell; score 1, 1–3 dots per cell (visible at 20–40×); score 2, 4–10 dots per cell and no or very few dot clusters (visible at 20–40×); score 3, >10 dots per cell and <10% positive cells have dot clusters (visible at 20×); score 4, >10 dots per cell and >10% positive cells have dot clusters (visible at 20×) (Figure 1). The probes for *UBC* (ubiquitin C) and *dapB* (a bacterial gene) were used as the positive and negative control, respectively. Samples were considered adequate when the *UBC* mRNA signals were easily visible under a 10x objective lens and the *dapB* signal was not visible.

### IHC and SISH

**Figure 2:**
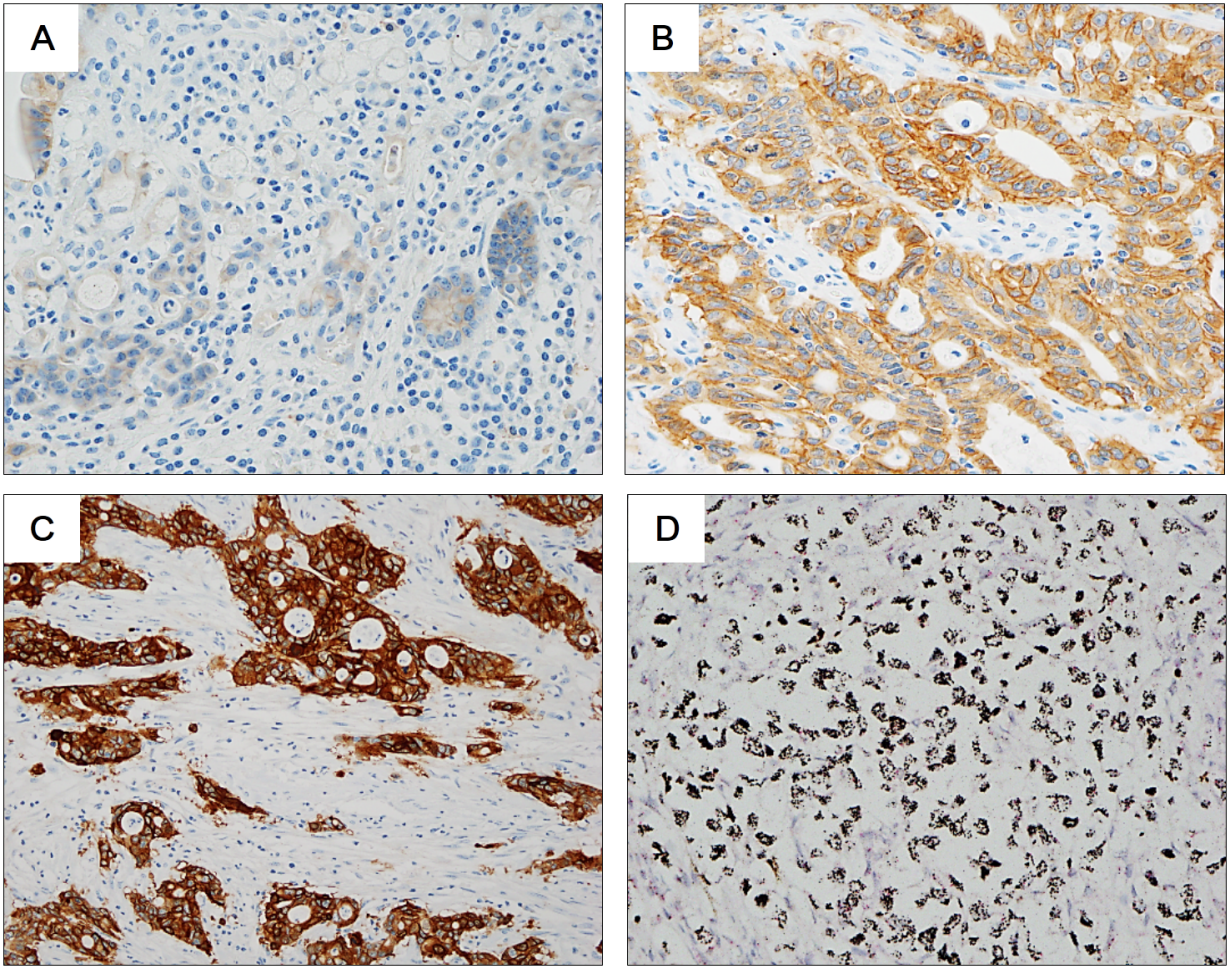
Representative figures of MET immunohistochemistry (IHC) and silver in situ hybridization. (A) IHC score 1, (B) IHC score 2, (C) IHC score 3, and (D) gene amplification (original magnification: ×400).

Immunohistochemical staining for MET was performed with anti-total MET (SP44) rabbit monoclonal primary antibodies (Ventana Medical Systems, Tucson, AZ, USA). An automatic immunostainer (BenchMark XT, Ventana Medical Systems) was used according to the manufacturer’s instructions. MET immunostaining was scored with the HercepTest scoring guidelines for GC (DAKO, Glostrup, Denmark): score 0, no membrane staining or membrane staining in <10% of tumor cells; score 1, faint/barely perceptible partial membrane staining in >10% of tumor cells; score 2, weak to moderate staining of the entire membrane in >10% of tumor cells; score 3, strong staining of the entire membrane in >10% of tumor cells (Figure 2).

Dual-color SISH assay was performed with INFORM MET DNA probe and INFORM Chromosome 7 probe (Ventana Medical Systems) on a Ventana BenchMark XT following the manufacturer’s protocols. Signals were enumerated in 40 tumor nuclei per core, and *MET* gene status was classified into 6 groups using the University of Colorado Cancer Center criteria for epidermal growth factor receptor gene [21] (Figure 2).

### Statistical analysis

The χ^2^ test or Fisher’s exact test was used to test the association between MET status and clinicopathologic factors. The Student’s *t*-test was used to compare means of continuous variables. The Spearman correlation test was used to assess the relationship between RNA ISH results and IHC or SISH results. The Kaplan-Meier method was used to estimate overall survival (OS), and OS differences between the groups with different MET status were compared by using the log-rank test. Multivariate survival analysis was performed using the Cox proportional hazards ratio model. Data analysis was conducted by using SPSS version 20.0 (SPSS, Chicago, IL, USA), and the results were considered significant when *P*<.05.

## Results

### 1. *MET* mRNA status assessed by RNA ISH

Of 535 primary tumors, 391 (73.1%), 87 (16.3%), 38 (7.1%), 12 (2.2%) and 7 (1.3%) showed RNA ISH score 0, 1, 2, 3, and 4, respectively. When we compared the results of RNA ISH with clinicopathologic data including survival, and analyzed results of RNA ISH with IHC and SISH data, the groups of score 3 and 4 showed distinct features. Therefore, we regarded the score 3 and 4 as high-*MET* mRNA group, and total 19 cases (3.5%) belonged to high-*MET* mRNA.

High-*MET* mRNA was associated with older age (*P* = .002), larger tumor size (*P* = .006), LN metastasis (*P* = .014), lymphatic invasion (*P*<.001), increased number of metastatic lymph nodes (*P*<.001), distant metastasis (*P* = .001), and higher TNM stage (*P*<.001) when compared to low-*MET* mRNA (Table 2). However, high-*MET* mRNA did not show any association with gender, tumor location, Lauren classification, and invasion depth (Table 2).

**Table 2:** Clinicopathologic characteristics of gastric carcinoma patients according to *MET* mRNA expression status.

Characteristics	*MET* mRNA status by RNA ISH		
	Low-*MET* mRNA	High-*MET* mRNA	*P*-value
	(score 0–2)	(score 3–4)	
	n = 516 (96.4%)	n = 19 (3.6%)	
Mean age, y	57.9	70	.002
Mean tumor size, cm	5.64	7.62	.006
Gender, n (%)			.639
Male	354 (69.6)	14 (73.7)	
Female	162 (31.4)	5 (26.3)	
Lauren classification, n (%)			.832
Intestinal	230 (44.6)	8 (42.1)	
Diffuse/mixed	286 (55.4)	11 (57.9)	
Tumor invasion, n (%)			.392
EGC	113 (21.9)	2 (10.5)	
AGC	403 (78.1)	17 (89.5)	
LN metastasis, n (%)			.014
Absent	277 (41.5)	3 (15.8)	
Present	391 (58.5)	16 (84.2)	
Distant metastasis, n (%)			.001
Absent	612 (91.6)	12 (63.2)	
Present	56 (8.4)	7 (36.8)	
TNM stage, n (%)			<.001
I	191 (28.6)	1 (5.3)	
II	201 (30.1)	3 (15.8)	
III	220 (32.9)	8 (42.1)	
IV	56 (8.4)	7 (36.8)	

Abbreviations: AGC, advanced gastric carcinoma; EGC, early gastric carcinoma; ISH, in situ hybridization; LN, lymph node; TNM, Tumor-Node-Metastasis.

Of 199 synchronous metastatic LNs, 119 (59.8%), 45 (22.6%), 22 (11.1%), 4 (2.0%) and 9 (4.5%) showed RNA ISH score 0, 1, 2, 3, and 4, respectively. Therefore, 13 cases (6.5%) were high-*MET* mRNA. Among 199 pairs of primary and metastatic lesions, 186 (93.5%) showed concordant *MET* mRNA status and 13 (6.5%) did not. Of these 13 discordant cases, negative conversion was found in 50% (7/14) of high-*MET* mRNA primary tumors, and positive conversion was found in 3.2% (6/185) of low-*MET* mRNA primary tumors (Table 3).

**Table 3:** Comparison of *MET* mRNA status between primary tumors and synchronous metastatic lymph nodes.

	Primary tumor		
	Low-*MET*mRNA	Low-*MET*mRNA	High-*MET*mRNA
	(score 0–1)	(score 2)	(score 3–4)
Metastatic lymph node			
Low-*MET* mRNA (score 0–1)	145	13	6
Low-*MET* mRNA (score 2)	17	4	1
High-*MET* mRNA (score 3–4)	3	3	7

### 2. MET protein and GCN status assessed by IHC and SISH

Using IHC, 236 (44.1%), 171 (32%), 113 (21.1%) and 15 (2.8%) primary tumors were scored 0, 1, 2 and 3, respectively. IHC score 3 showed distinct clinicopathologic features, and this MET overexpression group was significantly associated with older age (*P* = .005), larger tumor size (*P* = .009), invasion depth (*P* = .05), LN metastasis (*P* = .018), lymphatic invasion (*P* = .026), increased number of metastatic lymph nodes (*P*<.001), distant metastasis (*P* = .007), and higher TNM stage (*P* = .001). However, MET overexpression did not show any association with gender, tumor location, and Lauren classification (Table S1).

Of 199 synchronous metastatic LNs, 46 (23.1%), 92 (46.2%), 46 (23.1%) and 15 (7.5%) showed IHC score 0, 1, 2 and 3, respectively. Among 199 pairs of primary and metastatic lesions, 187 (94.0%) showed concordant MET protein status and 12 (6.0%) did not. Of these 12 discordant cases, negative conversion was found in 36.4% (4/11) of primary tumors with MET overexpression, and positive conversion was found in 4.3% (8/188) of primary tumors without MET overexpression.

Using SISH, *MET* GA was observed in 2.6% (14/535) of primary tumors. *MET* GA showed significant association with larger tumor size (*P* = .038), lymphatic invasion (*P* = .009), increased number of metastatic lymph nodes (*P*<.001), distant metastasis (*P* = .005), and higher TNM stage (*P* = .002). However, *MET* GA did not show any association with gender, tumor location, Lauren classification, invasion depth and LN metastasis (Table S2).

### 3. Correlation of MET status evaluated by RNA ISH, IHC and SISH

The correlation between *MET* mRNA and protein status assessed by RNA ISH and IHC is presented in Table 4. In 535 primary tumors, there was a positive correlation between *MET* mRNA and protein expression (r = 0.398, *P*<.001). All 7 cases with RNA ISH score 4 showed MET protein overexpression. Among 12 cases with RNA ISH score 3, 5 cases (41.7%) showed IHC score 3. The cases with RNA ISH score 2 exhibited variable IHC scores. The cases with RNA ISH score 0 or 1 did not show MET protein overexpression except for 1 case. Among 10 cases showing discrepancy (i.e., positive in RNA ISH and negative in IHC or vice versa), 8 showed IHC score 2 or RNA ISH score 2. In 199 metastatic LNs, there was a good positive correlation between *MET* mRNA and protein expression (r = 0.462, *P*<.001) (Table S3).

**Table 4:** Correlation of MET mRNA assessed by RNA in situ hybridization with protein and gene copy number assessed by immunohistochemistry and silver in situ hybridization.

	RNA ISH score, n (%)				
	0 (n = 391)	1 (n = 87)	2 (n = 38)	3 (n = 12)	4 (n = 7)
IHC score					
0 (n = 236)	205 (52.4)	26 (29.9)	5 (13.2)	0 (0)	0 (0)
1 (n = 171)	131 (33.5)	26 (29.9)	13 (34.2)	1 (8.3)	0 (0)
2 (n = 113)	54 (13.8)	35 (40.2)	18 (47.4)	6 (50.0)	0 (0)
3 (n = 15)	1 (0.3)	0 (0)	2 (5.3)	5 (41.7)	7 (100)
SISH					
DS (n = 221)	190 (48.6)	27 (31.0)	4 (10.5)	0 (0)	0 (0)
LT (n = 130)	96 (24.6)	22 (25.3)	12 (31.6)	0 (0)	0 (0)
HT (n = 1)	1 (0.3)	0 (0)	0 (0)	0 (0)	0 (0)
LP (n = 111)	75 (19.2)	23 (26.4)	10 (26.3)	3 (25.0)	0 (0)
HP (n = 58)	29 (7.4)	15 (17.2)	10 (26.3)	3 (25.0)	1 (14.3)
GA (n = 14)	0 (0)	0 (0)	2 (5.3)	6 (50.0)	6 (85.7)

Abbreviations: DS, disomy; GA, gene amplification; HP, high polysomy; HT, high trisomy; IHC, immunohistochemistry; ISH, in situ hybridization; LP, low polysomy; LT, low trisomy; SISH, silver in situ hybridization.

In addition, there was a positive correlation between *MET* mRNA expression and *MET* GCN (r = 0.345; *P*<.001) (Table 4). Among the 7 cases with RNA ISH score 4, 6 (85.7%) showed *MET* GA and only one case showed HP (14.3%). The 12 cases with RNA ISH score 3 showed GA (50%) or polysomy (50%) by SISH. The cases with RNA ISH score 2 showed various SISH patterns including GA (5.3%). None of the cases with RNA ISH score 0 or 1 showed GA.

Table 5 summarizes the results of RNA ISH, IHC and SISH of primary GC. Among the 535 cases, 513 cases (95.9%) were negative by both RNA ISH and IHC, and only 22 cases (4.1%) showed positive results by either RNA ISH or IHC. These 22 cases exhibited *MET* GA (54.5%) or polysomy (45.5%) by SISH, and disomy or trisomy was never observed. In terms of SISH, among the 14 cases showing GA, 11 cases (78.6%) exhibited high expression of both mRNA and protein. Among the 58 cases showing HP, however, only 7 cases (12.1%) exhibited high expression of either mRNA or protein. Among the 111 cases showing LP, only 3 cases (2.7%) exhibited high mRNA expression. All 352 cases showing disomy or trisomy exhibited negative results by both RNA ISH and IHC.

**Table 5:** Simultaneous comparison of MET status evaluated by RNA in situ hybridization, immunohistochemistry and silver in situ hybridization.

RNA ISH scores	IHC scores	SISH patterns, n (%)			
		Non-GA (n = 521)			GA (n = 14)
		DS, TS (n = 352)	LP (n = 111)	HP (n = 58)	
0/1	0/1	291 (82.7)	73 (65.8)	24 (41.4)	0 (0)
0/1	2	45 (12.8)	25 (22.5)	19 (32.8)	0 (0)
0/1	3	0 (0)	0 (0)	1 (1.7)	0 (0)
2	0/1	9 (2.6)	6 (5.4)	3 (5.2)	0 (0)
2	2	7 (2.0)	4 (3.6)	5 (8.6)	2 (14.3)
2	3	0 (0)	0 (0)	2 (3.4)	0 (0)
3/4	0/1	0 (0)	0 (0)	1 (1.7)	0 (0)
3/4	2	0 (0)	3 (2.7)	2 (3.4)	1 (7.1)
3/4	3	0 (0)	0 (0)	1 (1.7)	11 (78.6)

Abbreviations: DS, disomy; GA, gene amplification; HP, high polysomy; IHC, immunohistochemistry; ISH, in situ hybridization; LP, low polysomy; SISH, silver in situ hybridization; TS, trisomy.

### 4. Prognostic implications of MET status in primary and metastatic lesions

**Figure 3:**
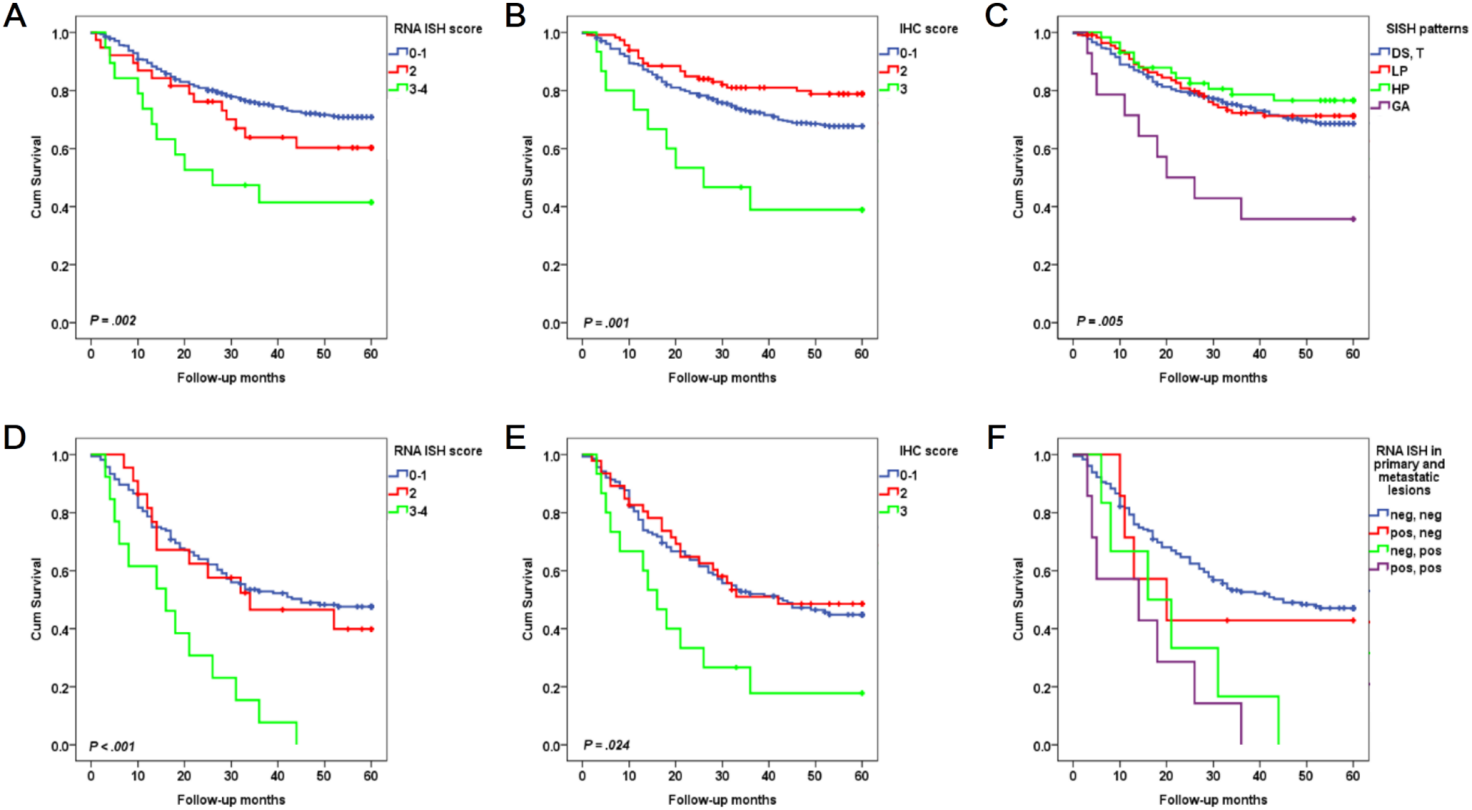
Kaplan-Meier curves for overall survival (OS) according to MET status. In 535 primary GC, (A) high-*MET* mRNA was associated with poor OS compared to low-*MET* mRNA (*P* = .002), (B) MET overexpression was associated with poor OS compared to no overexpression (*P* = .001), and (C) *MET* gene amplification was associated with poor OS compared to no amplification (*P* = .005). In 199 metastatic lymph nodes, (D) high-*MET* mRNA was associated with poor OS compared to low-*MET* mRNA (*P*<.001), and (E) MET overexpression was associated with poor OS compared to no overexpression (*P* = .024). (F) In 199 matched primary tumors and metastatic LNs, concordantly positive and positive conversion groups were associated with poor OS compared to concordantly negative group (concordantly negative vs. negative conversion, *P* = .640; concordantly negative vs. positive conversion, *P* = .011; concordantly negative vs. concordantly positive, *P*<.001; concordantly positive vs. negative conversion, *P* = .137; concordantly positive vs. positive conversion, *P* = .382; negative conversion vs. positive conversion, *P* = .260).

High-*MET* mRNA in primary tumors or metastatic LNs was significantly associated with poor OS (primary tumors, *P* = .002, Figure 3A; metastatic LNs, *P*<.001, Figure 3D). In the primary tumors with low-*MET* mRNA, the patients with positive conversion showed worse OS than those with no conversion (*P* = .011, Figure 3F). In the primary tumors with high-*MET* mRNA, the patients with negative conversion showed better OS than those with no conversion, but there was no statistical significance (*P* = .137, Figure 3F).

MET overexpression in primary tumors or metastatic LNs was significantly associated with poor OS (primary tumors, *P* = .001, Figure 3B; metastatic LNs, *P* = .024, Figure 3E). In the primary tumors without MET overexpression, positive conversion trended toward prediction of poor OS, but there was no statistical significance (*P* = .393). In the primary tumors with MET overexpression, negative conversion showed a trend of better OS than no conversion, but it did not reach statistical significance (*P* = .132). In addition, *MET* GA in primary tumors was also significantly associated with poor OS (*P* = .005, Figure 3C).

In multivariate analysis, high-*MET* mRNA in metastatic LNs was an independent negative prognostic factor for OS, after adjusting for age (<60 y vs. ≥60 y), Lauren classification (intestinal type vs. diffuse or mixed type), and TNM stage (I–II vs. III–IV). The hazard ratio was 2.27 (*P* = .007) (Table 6). However, MET overexpression in metastatic LNs was not a statistically significant prognostic factor by multivariate analysis, although the hazard ratio was 1.76 (*P* = .067). In addition, *MET* GA, high-*MET* mRNA or protein overexpression in primary tumor was not a statistically significant prognostic factor by multivariate analysis (data not shown).

**Table 6:** Univariate and multivariate Cox proportional hazards ratio model for the predictors of overall survival in gastric carcinoma (n = 199).

Characteristics	Univariate analysis		Multivariate analysis	
	HR (95% CI)	*P-value*	HR (95% CI)	*P-value*
Age	1.47 (1.00–2.15)	.05	1.55 (1.05–2.30)	.029
(<60 y vs. ≥60 y)				
Lauren classification	1.36 (0.93–2.00)	.117	1.34 (0.90–1.98)	.149
(Intestinal vs. diffuse/mixed)				
TNM stage	12.8 (4.06–40.4)	<.001	11.8 (3.73–37.4)	<.001
(Stage I–II vs. III–IV)				
RNA ISH score of metastatic LN	3.18 (1.77–5.70)	<.001	2.27 (1.26–4.09)	.007
(0–2 vs. 3–4)				

Abbreviations: CI, confidence interval; HR, hazard ratio; ISH, in situ hybridization; LN, lymph node.

## Discussion

In the present study, we demonstrated that high *MET* mRNA expression was significantly associated with adverse clinicopathologic features and poor prognosis in a large series of GC patients using RNA ISH method. In addition, RNA ISH results were well correlated with those of SISH and IHC. The previous studies evaluating *MET* mRNA levels in GC used the Northern blot assay [8], [22] or reverse transcription-polymerase chain reaction (RT-PCR) [8], [14], [23]–[25]. However, most of the studies had small sample sizes, and only a few of them investigated its clinical implications [22], [24] or performed comparison with DNA or protein status [14], [25]: Kuniyasu et al. firstly studied MET mRNA expression using the Northern-blot analysis, and they reported that expression of 6.0-kb transcript was closely correlated with tumor stage and LN metastasis [22]. Amemiya et al. reported that Stage IV GC patients with liver metastasis showed higher MET expression at both mRNA and protein levels than stage IV GC patients without liver metastasis using RT-PCR and IHC [24].

Recently, we reported that high levels of *HER2* mRNA was well correlated with protein overexpression and GA by comparing the results of 4 different in situ-based methodologies (RNA ISH, IHC, FISH, and SISH) in 211 GC cases [20]. Likewise, in this study for MET status, the results of RNA ISH showed fairly good correlation with those of IHC and SISH. These results support that RNA ISH can be a reliable assay for FFPE tissue samples, although further validation studies are needed.

We demonstrated that *MET* GA, high *MET* mRNA and protein overexpression evaluated by SISH, RNA ISH and IHC were highly concordant, and high MET status at the DNA, mRNA, and protein were significantly associated with poor prognosis. These findings support that MET overexpression is mainly due to increased *MET* GCN and this mechanism contributes to aggressive behavior of *MET* oncogene-addicted GC. Nevertheless, there were some cases showing inconsistency among the *MET* GCN, mRNA and protein levels. We speculate that technical problems (e.g., sensitivity and specificity of the probe or antibody, and poor mRNA quality of FFPE tissues) and intratumoral heterogeneity of MET status may be the main causes of this discrepancy. However, some biological mechanisms can also be related to this discrepancy. For example, MET overexpression without GA can occur through transcriptional activation via HGF-dependent autocrine/paracrine loops or other signaling pathways [23], [26]. On the contrary, *MET* GA may not increase the gene product. Asaoka et al. reported that a few GC cell lines harboring *MET* GA expressed the protein as same level as other cell lines without GA, but their tyrosine residues at the kinase domain were more phosphorylated [27]. The mechanisms of MET activation and the role of HGF in GC remain to be elucidated.

It is well known that *MET* plays a role in metastatic progression of cancer. Several studies showed that *MET* GA or overexpression was associated with LN metastasis [7], [13], [22] or distant metastasis [13], in GC patients. In addition, it was shown that the administration of MET inhibitor reduced peritoneal dissemination of GC in a xenograft model [23]. Furthermore, Di Renzo et al. found that cancer cells carrying *MET* activating mutations were selected during metastatic spread of head and neck squamous cell carcinomas by comparing the gene sequence between primary tumor and metastatic lymph node [28]. However, direct comparison of MET status between primary tumor and metastasis has not been performed in a large series of GC. When we compared the MET expression status between 199 matched primary tumors and metastatic LNs, and the overall concordance was 93.5% and 94% by RNA ISH and IHC, respectively. These results suggest that MET expression status of GC is relatively constant during metastasis to regional LNs. However, positive conversion of *MET* mRNA status was significantly associated with poor prognosis by univariate and multivariate analysis. Therefore, these results suggest that the evaluation of MET status in metastatic lesions may be important to predict prognosis and to identify additional candidates for MET-targeted therapy.

In situ-based RNA analysis has several advantages over the ‘grind and bind’ analysis such as RT-PCR [19] and is applicable for both clinical practice and retrospective research. Moreover, RNA ISH is more favorable than IHC when there is no suitable antibody or when the target molecule is the secreted protein. In regard to *MET*, this advantage can be useful because the HGF-producing cells can be visualized in a tissue section using the HGF probe. However, vulnerable mRNA stability during the tissue processing and higher cost than that of IHC are the disadvantages of RNA ISH method. We hope that further technical improvement will resolve these limitations.

In this retrospective study, *MET* mRNA status evaluated by RNA ISH is well correlated with protein and GCN assessed by IHC and SISH, respectively, and *MET* GA is highly concordant with high expression of either mRNA or protein. In survival analysis, high expression of *MET* mRNA in primary or metastatic lesions, and positive conversion of *MET* mRNA status are significantly associated with poor prognosis. In addition, *MET* mRNA status in metastatic LNs is an independent prognostic factor by multivariate analysis. Our findings indicate that *MET* mRNA can be an alternative marker to identify the *MET* oncogene-addicted GC.

## Supporting Information

Table S1 (DOCX)

Table S2 (DOCX)

Table S3 (DOCX)
