# Supplementary material for: Analysis of MET mRNA Expression in Gastric Cancers Using RNA In Situ Hybridization Assay: Its Clinical Implication and Comparison with Immunohistochemistry and Silver In Situ Hybridization
Source: PLoS One. 2014 Nov 3;9(11):e111658. doi: 10.1371/journal.pone.0111658 (PMC4218795; doi:10.1371/journal.pone.0111658)
Supplement: Table S1 — (DOCX) [file pone.0111658.s001.docx]

**Table S1. Clinicopathologic characteristics of gastric carcinoma patients according to MET protein expression status**

| Characteristics | MET protein status by IHC | | |
| --- | --- | --- | --- |
|  | No overexpression  (score 0-2) | Overexpression  (score 3) | *P*-value |
|  | n=520 (97.2%) | n=15 (2.8%) |  |
| Mean age, y | 58.0 | 67.2 | .005 |
| Mean tumor size, cm | 5.65 | 7.76 | .009 |
| Gender, n (%) |  |  | .411 |
| Male | 356 (68.5) | 12 (80.0) |  |
| Female | 164 (31.5) | 3 (20.0) |  |
| Lauren classification, n (%) |  |  | .863 |
| Intestinal | 231 (44.4) | 7 (46.7) |  |
| Diffuse/mixed | 289 (55.6) | 8 (53.3) |  |
| Tumor invasion, n (%) |  |  | .05 |
| EGC | 115 (22.1) | 0 (0) |  |
| AGC | 405 (77.9) | 15 (100) |  |
| LN metastasis, n (%) |  |  | .018 |
| Absent | 229 (44.0) | 2 (13.3) |  |
| Present | 291 (56.0) | 13 (86.7) |  |
| Distant metastasis, n (%) |  |  | .007 |
| Absent | 477 (91.7) | 10 (66.7) |  |
| Present | 43 (8.3) | 5 (33.3) |  |
| TNM stage, n (%) |  |  | .001 |
| I | 170 (32.7) | 0 (0) |  |
| II | 139 (26.7) | 3 (20.0) |  |
| III | 168 (32.3) | 7 (46.7) |  |
| IV | 43 (8.3) | 5 (33.3) |  |

Abbreviations: AGC, advanced gastric carcinoma; EGC, early gastric carcinoma; IHC, immunohistochemistry; LN, lymph node; TNM, Tumor-Node-Metastasis
