# Supplementary material for: Analysis of MET mRNA Expression in Gastric Cancers Using RNA In Situ Hybridization Assay: Its Clinical Implication and Comparison with Immunohistochemistry and Silver In Situ Hybridization
Source: PLoS One. 2014 Nov 3;9(11):e111658. doi: 10.1371/journal.pone.0111658 (PMC4218795; doi:10.1371/journal.pone.0111658)
Supplement: Table S2 — (DOCX) [file pone.0111658.s002.docx]

**Table S2. Clinicopathologic characteristics of gastric carcinoma patients according to *MET* gene copy number status**

| Characteristics | *MET* GCN by SISH | | |
| --- | --- | --- | --- |
|  | No amplification | Amplification | *P*-value |
|  | n=521 (97.4%) | n=14 (2.6%) |  |
| Mean age, y | 58.1 | 64.4 | .063 |
| Mean tumor size, cm | 5.67 | 7.39 | .038 |
| Gender, n (%) |  |  | .772 |
| Male | 359 (68.9) | 9 (64.3) |  |
| Female | 162 (31.1) | 5 (35.7) |  |
| Lauren classification, n (%) |  |  | .901 |
| Intestinal | 232 (44.5) | 6 (42.9) |  |
| Diffuse/mixed | 289 (55.5) | 8 (57.1) |  |
| Tumor invasion, n (%) |  |  | .321 |
| EGC | 114 (21.9) | 1 (7.1) |  |
| AGC | 407 (78.1) | 13 (92.9) |  |
| LN metastasis, n (%) |  |  | .264 |
| Absent | 227 (43.6) | 4 (28.6) |  |
| Present | 294 (56.4) | 10 (71.4) |  |
| Distant metastasis, n (%) |  |  | .005 |
| Absent | 478 (91.7) | 9 (64.3) |  |
| Present | 43 (8.3) | 5 (35.7) |  |
| TNM stage, n (%) |  |  | .002 |
| I | 168 (32.2) | 2 (14.3) |  |
| II | 141 (27.1) | 1 (7.1) |  |
| III | 169 (32.4) | 6 (42.9) |  |
| IV | 43 (8.3) | 5 (35.7) |  |

Abbreviations: AGC, advanced gastric carcinoma; EGC, early gastric carcinoma; GCN, gene copy number; LN, lymph node; SISH, silver in situ hybridization; TNM, Tumor-Node-Metastasis
