# Supplementary material for: Analysis of MET mRNA Expression in Gastric Cancers Using RNA In Situ Hybridization Assay: Its Clinical Implication and Comparison with Immunohistochemistry and Silver In Situ Hybridization
Source: PLoS One. 2014 Nov 3;9(11):e111658. doi: 10.1371/journal.pone.0111658 (PMC4218795; doi:10.1371/journal.pone.0111658)
Supplement: Table S3 — (DOCX) [file pone.0111658.s003.docx]

**Table S3. Correlation between *MET* mRNA and protein assessed by RNA in situ hybridization and immunohistochemistry in 199 metastatic lymph nodes**

|  | RNA ISH score, n (%) | | | | |
| --- | --- | --- | --- | --- | --- |
|  | 0 | 1 | 2 | 3 | 4 |
| IHC score |  |  |  |  |  |
| 0 | 37 (31.1) | 7 (15.6) | 2 (9.1) | 0 (0) | 0 (0) |
| 1 | 62 (52.1) | 23 (51.1) | 6 (27.3) | 1 (25.0) | 0 (0) |
| 2 | 20 (16.8) | 14 (31.1) | 11 (50.0) | 1 (25.0) | 0 (0) |
| 3 | 0 (0) | 1 (2.2) | 3 (13.6) | 4 (50.0) | 9 (100) |

Abbreviations: IHC, immunohistochemistry; ISH, in situ hybridization
